# Surgical Management for a Rare Pedunculated Left Ventricular Apical Lipoma: A Case Report and Review of Literature

**DOI:** 10.3389/fcvm.2021.723975

**Published:** 2021-12-10

**Authors:** Long Song, Chukwuemeka Daniel Iroegbu, Jinfu Yang, Chengming Fan

**Affiliations:** Department of Cardiovascular Surgery, The Second Xiangya Hospital, Central South University, Changsha, China

**Keywords:** cardiac surgery, computed tomography, left ventricular lipoma, primary cardiac tumors, transthoracic echocardiography

## Abstract

Cardiac lipomas, though extremely rare, are encapsulated tumors composed primarily of mature fat cells. Despite their benign character, cardiac lipomas can cause life-threatening complications by rapid growth. Cardiac lipomas, which are frequently located in the left ventricle (LV) or right atrium, can originate either from the subendocardium, subpericardium, or the myocardium. They are usually asymptomatic and carry a good prognosis during long-term follow-up; however, published reports show that untreated cardiac lipomas may be fatal when they cause arrhythmic or obstructive symptoms. In addition, several surgical options have been reported to obtain an appropriate operative view following poor visualization, primarily when tumors are located in the LV. Herein, we present a case of a pedunculated LV apical lipoma in a symptomatic patient successfully managed by surgical resection. We also discuss diagnostic modalities in surgical planning and the choice of surgical approach.

## Introduction

Around 75% of primary cardiac tumors are benign, with an incidence of 0.2–0.4% in autopsy series (1). Cardiac lipomas, though extremely rare with a reported incidence ranging from 2.9 to 8% among all benign cardiac tumors (1) and 10% of all neoplasm's of the heart (2), are encapsulated tumors composed primarily of mature fat cells. Cardiac lipomas are 50 times less common than myxomas and usually present in combination with lipomas of other organs. These tumors are often asymptomatic and usually detected incidentally, mostly during autopsies. In symptomatic patients, the diagnosis can easily be made by echocardiography, computed tomography (CT), or magnetic resonance imaging (MRI).

The etiology of lipomas remains uncertain; however, an association with chromosome 12 gene rearrangements has been established in solitary lipoma cases with an abnormality in the HMGA2-LPP fusion gene (3). These tumors are typically found in adult patients in their fifth or sixth decade of life but can affect patients of all ages, including both sexes (4). Lipomas of the heart can be intracavitary, epimyocardial, or intramyocardial and may be located in the atria or ventricles. Although lipomas are benign, these tumors can cause cardiac chamber(s) compression, chest tightness, arrhythmias, obstructive symptoms, and other life-threatening complications when the tumor increases in size (5). Conservative management includes clinical observation and, in some cases, anticoagulation. Surgical management includes resection of the mass, which is considered curative. Whether one approach is superior to the other is unknown given the lack of data and the tumor's rarity.

Herein, we present a case of a pedunculated left ventricular (LV) apical lipoma in a symptomatic patient successfully managed by surgical resection. We also discuss diagnostic modalities in surgical planning and the choice of surgical approach.

## Case Presentation

A 52-year-old male was referred to our institution complaining of chest tightness and dyspnea on exertion the last few years, which only progressively worsened the last few months (NYHA III) with palpitations and edema of the lower limbs. The patient had neither previous cardiovascular antecedents and denied familial history. He also denied medication use with only occasional smoking and alcohol intake. The patient's body temperature was normal on admission, 36.8°C with blood pressure 126/82 mmHg, and a radial pulse rate of 83 bpm. The patient body mass index was 24.2.

Complete blood count, basic metabolic profile, B-type natriuretic peptide, and troponins were also within normal limits. The biochemical investigation showed no alteration in the following serum levels: thyrotropic hormone, potassium, alkaline phosphatase, glycemia, sodium, hyaluronic acid, urea, total bilirubin, and creatinine. Hemoglobin level was 103 g/l, hematocrit level was 31.4%, while serum leukocyte and platelet levels were 6.61 × 10^9^/L and 214 × 10^9^/L, respectively.

Physical examination and vital signs were unremarkable. The patient had good psychomotor development and no neurological signs or nystagmus. On cardiovascular examination, the patient had poor peripheral perfusion, asymmetric pulses, postural hypotension, peripheral lower limb edema, and a mild ejective cardiac murmur at the mitral region. ECG showed non-specific T-wave abnormalities in the lateral leads. The lungs were clear to auscultation, while other body systems showed no abnormalities on physical examination.

### Radiological Investigations

**Figure 1:**
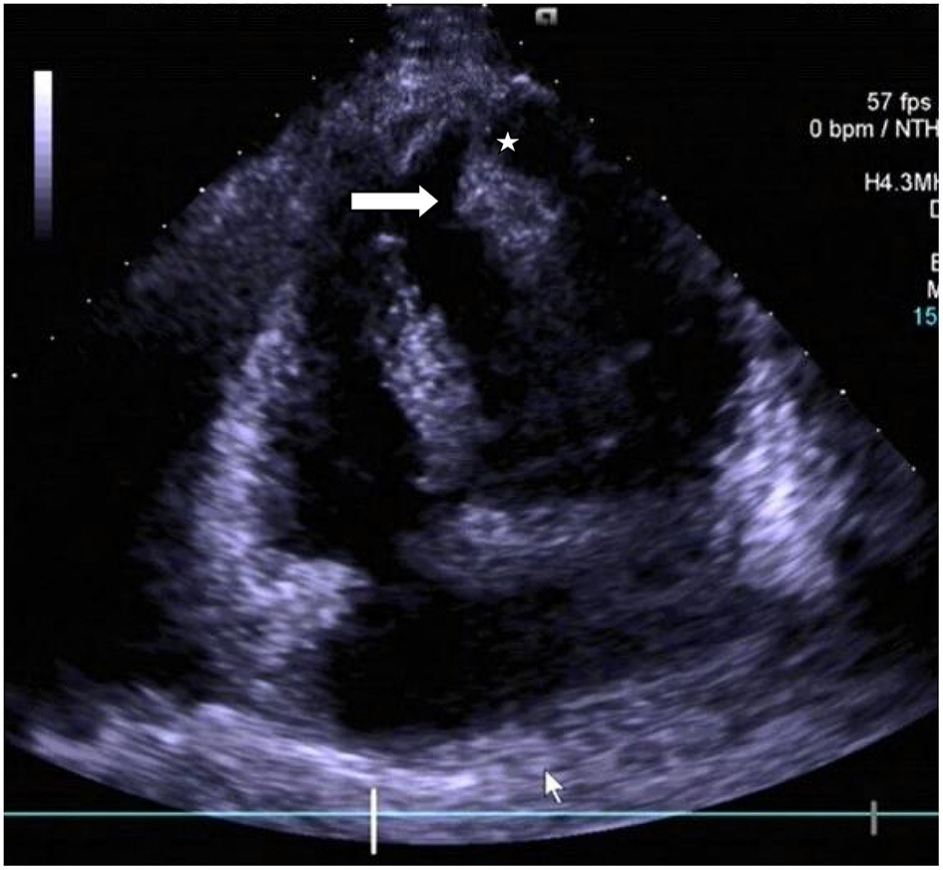
Transthoracic echocardiogram revealing the presence of a pedunculated lobular mass [peduncle (asterisk); mass (arrow head)] within the left ventricle.

**Figure 2:**
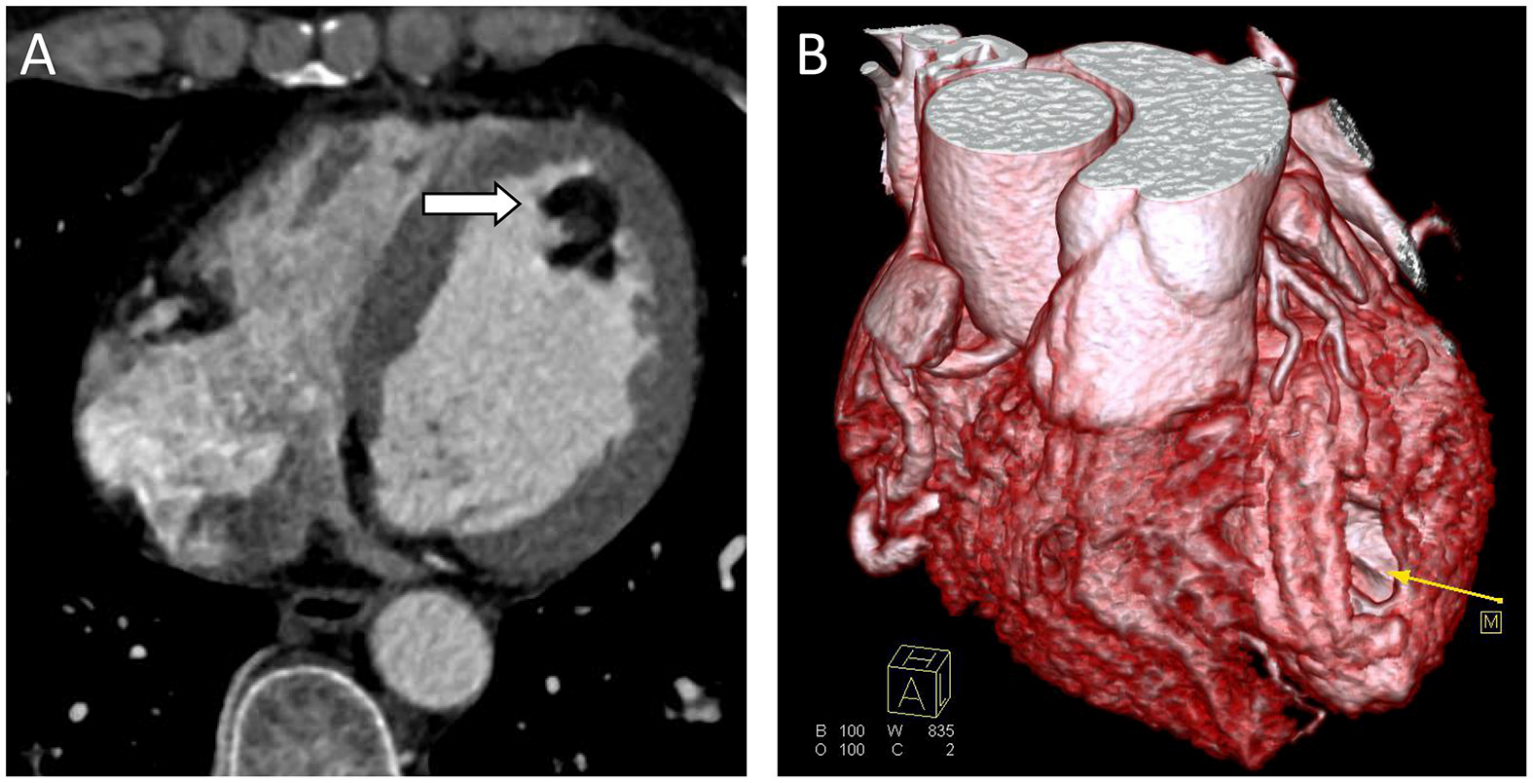
Computed Tomography (CT) scans. **(A)** CT scan showing a well-defined hypodense tumor in the left ventricle (arrow). **(B)** CTA 3D reconstruction; hypodense tumor (yellow arrow).

Chest radiograph showed a moderately enlarged cardiac silhouette with increased cardiothoracic ratio and no signs of pulmonary congestion. Transthoracic echocardiogram (TTE) examination showed the following characteristics: aorta 34 mm, left atrium 35 mm, LV cavity 51 mm (end-diastolic diameter), and a hyperechoic mass 30 × 27 mm located on the left side of the heart (middle-apical region), occupying almost 2/3 of the LV with blood supply from the left anterior descending (LAD) coronary artery (Figure 1). The vast mass caused a marked dilatation of the LV with mild to moderate LV outflow tract (LVOT) obstruction and decreased ventricular ejection. In addition, the apical and diaphragmatic part of the tumor was mildly calcified. CT scan of the chest confirmed a mass measuring 28 × 32 mm (Figure 2). The hyperechoic mass was highly mobile, with a peduncle measuring ~4 cm in diameter, adhering to the apical diaphragmatic surface of the LV free wall and papillary muscle.

### Differential Diagnoses

The characteristics of the mass following clinical investigations were suggestive of a fat-containing lesion at the LV apex. Hence, the differential diagnoses were relatively limited to subendocardial fat collection, LV thrombus, lipomatous hypertrophy, fibroma, intracardiac varix, and lipoma. However, lipomatous hypertrophy primarily occurred in the interatrial septum and was therefore judged less likely given the location of the patient's mass. Nonetheless, other intracardiac masses such as teratomas, fibroma, and myxoma could not be initially excluded.

### Management

The patient was anticoagulated with apixaban as a “bridge” to definitive surgical therapy. The case and radiological reports were discussed in a multi-disciplinary heart team meeting, and the final consensus for a surgical resection was reached. Likewise, the case was also discussed with the patient. A unanimous decision was made to proceed with the surgical resection. Surgery was performed via median sternotomy, and cardiopulmonary bypass was established by aortic and two-stage single venous cannulation (mild hypothermia at 30°C). An aortic cross-clamp was applied, and myocardial protection was established with an antegrade infusion of cold Custodiol® HTK solution via the aortic root.

**Figure 3:**
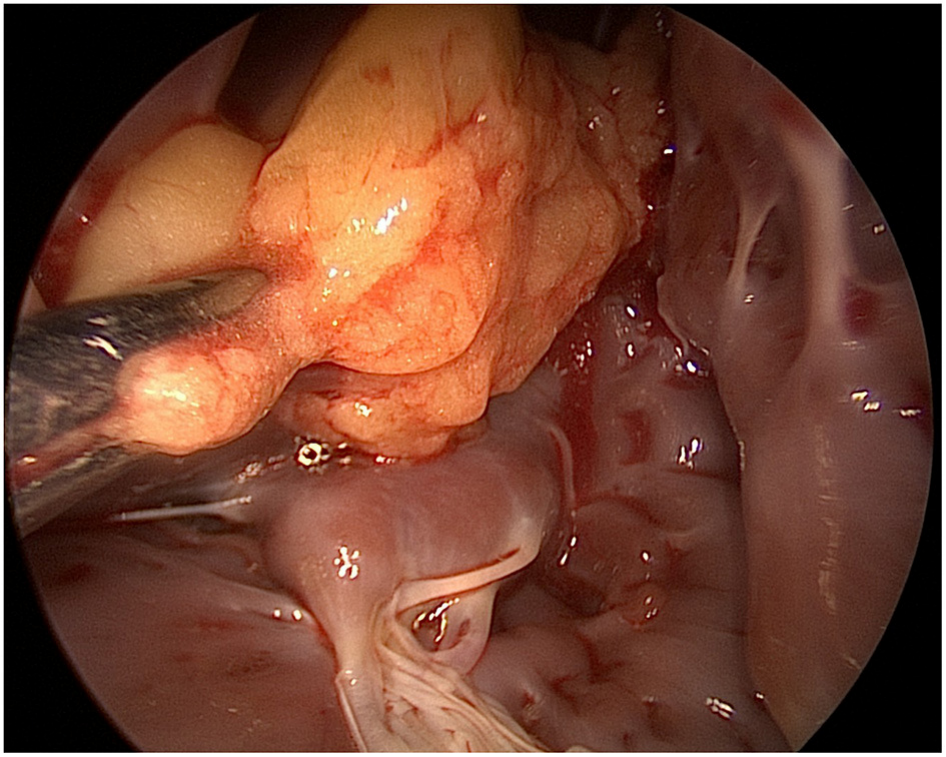
Mediastinoscope image via a left atriotomy showing a yellowish well-encapsulated mass within the left ventricle.

The left heart and the LV mass were approached via a left atriotomy approach. However, given the extensive and deeply located position of the mass at the LV apex with mild adherence to the LV free wall and papillary muscles, we inserted a mediastinoscope into the LV. The mediastinoscope provided a more detailed view than the usual valve retractors, providing a safe resection of the mass (Figure 3). The tumor looked like a lobulated fatty mass, which was well-encapsulated with a yellowish appearance. Under direct vision, the lipoma was resected using scissors, and the LV was rinsed and cleared with long laparoscopy forceps and continuous suction.

**Figure 4:**
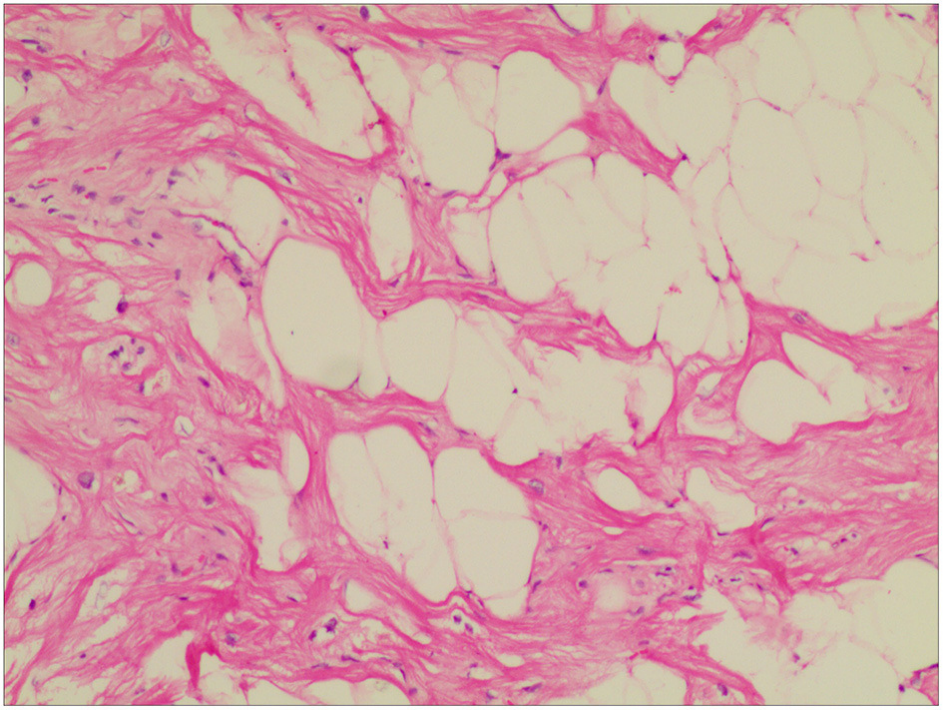
Histopathological examination of the excised mass showed mature adipocytes, consistent with lipoma accompanied by some small vessels and limited collagenous capsule. HandE staining, ×10.

The operation was uneventful. Hemostasis was achieved, and the chest was closed after placing one chest tube in the pericardium. The patient was discharged from the anesthetic recovery room on the 2nd post-operative day. Apixaban was discontinued; however, whether or not post-operative anticoagulation is needed is debatable. Intraoperative histopathologic examination of the excised specimen revealed fatty structure (mature adipose tissue) with areas of increased vascularity (Figure 4). The results were consistent with benign encapsulated lipoma. The post-operative course was also uneventful, and the patient was discharged from the hospital on the 6th post-operative day.

### Follow-Up Period

During the follow-up period, consecutive TTEs performed during 5-years on an outpatient care basis detected no intracardiac lesion and signs of recurrence; however, it showed moderate mitral insufficiency. Further evaluation showed arrhythmia (atrial fibrillation), mild intra-atrial and ventricular pressure increase, and poor apical LV wall motion (apical cap, anterior, and the lateral walls) with scarring (possibly from the inadequate or compromised LAD tributary). The LV ejection fraction was 54%.

## Discussion

Cardiac lipomas of the heart are sporadic tumors, accounting for 2–8% of all benign cardiac tumors (6, 7) and incidentally discovered during routine chest X-rays or cardiac investigations. Lipomas occur at an equal frequency in both genders (8), with the typical “age at presentation” between 40 and 60-years of age (9). Cardiac lipomas, particularly in the early stages, are usually indolent and asymptomatic and can originate either from the subendocardium, subpericardium, or myocardium and may be located more frequently in the right atrium, LV (7, 10), or both ventricular cavities (11). Symptoms are not characteristic. Generally, most cardiac lipomas are silent, with only a few situations where clinical symptoms manifest, depending on their location and size. Thus, angina, arrhythmia, and syncope are symptoms a patient may complain of on routine clinical visits. In the case herein, the left-sided heart failure symptoms were precipitated by the (fat-attenuating) pedunculated intracardiac mass obstructing the LVOT (Sun et al.).

Also, the dare issue with lipoma does not reside in its histological characteristics but its intracavitary component. Thus, the presence of cardiac lipoma becomes lethal when it occupies the LV cavity, resulting in obstruction and compression, which eventually causes peripheral embolism, LV failure, and rhythm and conduction disorders (12–14). The patient's symptoms in the presented case were those of heart failure with dyspnea and peripheral edemas. Given that lipomas are well-encapsulated, unlike myxomas with friable structures (15), the patient showed no evidence of embolus or stroke in his clinical history and medical records.

Nonetheless well-encapsulated, the uncertainty to patients with cardiac lesions (if a patient should receive “bridging” before) and its discontinuity remains debatable and unclear. Firstly, bridging anticoagulation refers to giving a short-acting blood thinner aimed at patients with arrhythmias (or potentially susceptible) or stroke risk. Also, the risks and benefits of “bridging” with a shorter-acting agent are still unclear. Though there is no single approach, bridging anticoagulation should be administered carefully. Another clinical issue is anticoagulant-specific. Whether bridging anticoagulation helps patients is also uncertain, given the existing contrasting opinions amongst physicians. However, with the lesion's rarity, unpredictable, and silent characteristics, the safe application of bridging could be a welcomed strategy, primarily when pivotal diagnostic modalities are not available or applied (as with the case herein). Direct oral anticoagulants such as dabigatran (factor Xa inhibitor; apixaban administered in the present case) have shorter half-lives, making them easier to discontinue or rapidly resume.

Though cardiac masses are in part discovered at autopsy (16–19) or incidentally following X-rays, diagnosing LV lipomas based on medical history, auscultation, and even X-ray alone is challenging. Hence, more dedicated imaging is warranted to characterize the mass. Furthermore, on cardiac imaging, cardiac lipomas are distinctively differentiated from adipo-sarcomas and lipomatous hypertrophy of the interatrial septum due to their encapsulated appearance, which was in the presented case (10).

Echocardiogram, which can non-invasively and directly observe tumor shape, size, influence on diastolic cardiac function, activity, relationship with adjacent tissues, and location, is currently the initial diagnostic test for patients with a suspected cardiac mass (10, 20, 21). However, TTE, in particular, is deficient in differentiating the various tissue characteristics, which was also so in the presented case (21). Nonetheless, transesophageal echocardiography (TEE), though helpful in visualizing masses not readily visible on TTE, was not used for the patient herein given that the apex is not adequately visualized in standard TEE views also distant from the transducer, thus limiting its resolution (22).

Subsequently, cardiac CT and cardiac magnetic resonance imaging (CMR) can be pursued to provide vital information concerning the extent of myocardial infiltration and tissue characterization and help differentiate a fat-containing lesion from a thrombus (7, 10). However, in the case herein, only cardiac CT was used. Notably, low-attenuation features with a density similar to fat on CT are pathognomonic for lipoma, as with the presented case. In addition, coronary arteriography though not used in the case herein, can provide crucial information by outlining the arterial supply and defining the coronary anatomy, which will be essential for the operating team.

The total surgical resection of lipomas with its pedicle is advocated (as with the presented case herein) to prevent a recurrence, whether the lipoma is intracardiac or subcutaneous in origin. However, there are significant surgical challenges and considerations when treating intracavitary lesions, such as (i) the maintenance of adequate atrioventricular valvular function, (ii) preserving an adequate portion of the ventricular myocardium, (iii) the satisfactory excision of the lesion from the LV without injuring it, and (iv) preserving the conduction system. Currently, given the rarity of the lesion, there are no evidence-based guidelines concerning their optimal treatment. Thus, conservative vs. surgical management decision solely relies on factors such as the patient's preference, symptomology, the lesion size and location, and surgical know-how.

Given the location of the lesion and the stalk deeply attached to the cardiac apex in the presented case, a left atriotomy approach was opted for, which, however, proved to be a surgical “pitfall.” Though the usual surgical approach for lipoma resection is through a median sternotomy, other surgical methods have been used with excellent results, particularly for apical and ventricular lesions. The reported surgical approaches include: (i) the LV tip approach; a similar incision used for LV aneurysms, which provides adequate exposure (preventing partial resection) and decreases the risk of embolization; (ii) video-assisted removal of cardiac lesions, which improves lighting inside the surgical field and avoids left ventriculotomy and the possible damage of surrounding structures (7, 23).

Though prompt and careful pre-operative qualitative diagnosis is necessary (7), definitive diagnoses for cardiac lipomas are made on post-operative pathological examination irrespective of the pre-operative diagnostic modalities used. In the presented case, cardiac lipoma following post-operative pathological examination was confirmed given the presence of mature, differentiated fatty tissue, with small amounts of fibrous connective tissue and blood vessels, which were surrounded by a fibrous membrane. Given that post-operative prognoses for lipomas are favorable, surgical managements are without complications such as LV systolic dysfunction and prolonged follow-up (as with the presented case), ventricular septal defects, and even death (8), primarily when performed late or with the use of a “non-suitable” surgical approach.

## Conclusion

Though there are no evidence-based guidelines concerning the optimal treatment of the disease given its rarity, the case herein exemplifies how early diagnosis, appropriate cardiac investigations, and surgical management of patients with cardiac lipoma should be individualized for favorable long-term outcomes. Also, with cases of apical cardiac lipoma, cardiac investigations should include coronary arteriography to define coronary anatomy and outline arterial supplies.

## Data Availability Statement

The original contributions presented in the study are included in the article/supplementary material, further inquiries can be directed to the corresponding author/s.

## Ethics Statement

The studies involving human participants were reviewed and approved by the Ethics Committee of the Second Xiangya Hospital of Central South University. The patients/participants provided their written informed consent to participate in this study.

## Author Contributions

CI drafted the manuscript. CF and LS designed the study. JY and CF revised the manuscript. LS was responsible for the collection of data or analysis. All authors have read and approved the final manuscript.

## Funding

This work was supported by the Science and Technology Innovation Program of Hunan Province (2021RC2106 to CF), Scientific Research Project of Hunan Provincial Health Commission (Grant No. 202104020921 to LS), and Natural Science Foundation of Hunan Province (Grant No. 2021JJ30951 to LS).

## Conflict of Interest

The authors declare that the research was conducted in the absence of any commercial or financial relationships that could be construed as a potential conflict of interest.

## Publisher's Note

All claims expressed in this article are solely those of the authors and do not necessarily represent those of their affiliated organizations, or those of the publisher, the editors and the reviewers. Any product that may be evaluated in this article, or claim that may be made by its manufacturer, is not guaranteed or endorsed by the publisher.

## References

[1] Colucci WS Schoen FJ Braunwald E. Primary Tumors of the Heart. Vol. 2. Philadelphia, PA: WB Saunders (1997).

[2] Hananouchi GI Goff WB II. Cardiac lipoma: six-year follow-up with MRI characteristics, and a review of the literature. Magn Reson Imaging. (1990) 8:825–8. 10.1016/0730-725X(90)90021-S 2266812

[3] Italiano A Ebran N Attias R Chevallier A Monticelli I Mainguene C. NFIB rearrangement in superficial, retroperitoneal, and colonic lipomas with aberrations involving chromosome band 9p22. Genes Chromosomes Cancer. (2008) 47:971–7. 10.1002/gcc.20602 18663748

[4] Tazelaar HD Locke TJ McGregor CG. Pathology of surgically excised primary cardiac tumors. Mayo Clin Proc. (1992) 67:957–65. 10.1016/S0025-6196(12)60926-4 1434856

[5] Conces DJJr Vix VA Tarver RD. Diagnosis of a myocardial lipoma by using CT. AJR Am J Roentgenol. (1989) 153:725–6. 10.2214/ajr.153.4.725 2773726

[6] Singh S Singh M Kovacs D Benatar D Khosla S Singh H. A rare case of a intracardiac lipoma. Int J Surg Case Rep. (2015) 9:105–8. 10.1016/j.ijscr.2015.02.024 25746952 PMC4392332

[7] Sun X Liu G Kim H Sun W. Left ventricular lipoma resected using thoracoscope-assisted limited sternotomy: a case report and literature review. Medicine. (2018) 97:e11436. 10.1097/MD.0000000000011436 30075509 PMC6081152

[8] Li YS. Surgical treatment of primary left ventricular lipoma: a case report and literature review. ChongQing Med. (2002) 31:167–9.30075509

[9] D'Souza J Shah R Abbass A Burt JR Goud A Dahagam C. Invasive Cardiac Lipoma: a case report and review of literature. BMC Cardiovasc Disord. (2017) 17:28. 10.1186/s12872-016-0465-2 28088193 PMC5237479

[10] Rocha RV Butany J Cusimano RJ. Adipose tumors of the heart. J Card Surg. (2018) 33:432–7. 10.1111/jocs.13763 29992619

[11] Ari H Ari S Goncu MT Koca V Bozat T. Biventricular lipoma (first case in literature). Int J Cardiol. (2011) 150:e98–100. 10.1016/j.ijcard.2010.02.058 20557958

[12] Akram K Hill C Neelagaru N Parker M. A left ventricular lipoma presenting as heart failure in a septuagenarian: a first case report. Int J Cardiol. (2007) 114:386–7. 10.1016/j.ijcard.2005.11.083 16624434

[13] Hayashi H Hidaka F Kiriyama T Sato H Takagi R Kumita S. A left ventricular lipoma diagnosed on three-dimensional electrocardiogram-gated cardiac computed tomography. Heart Vessels. (2008) 23:366–9. 10.1007/s00380-007-1037-2 18810588

[14] Lin HD Hsu PF Wu MH Leu HB Hsu TL. Images in cardiology: subaortic stenosis caused by left ventricular outflow tract lipoma. Clin Cardiol. (2006) 29:421. 10.1002/clc.4960290911 17007176 PMC6654320

[15] Censi S Squeri A Baldelli M Parizi ST. Ischemic stroke and incidental finding of a right atrial lipoma. J Cardiovasc Med. (2013) 14:905–6. 10.2459/JCM.0b013e328364bf8b 24149062

[16] Domoto S Nakano K Kodera K Sasaki A Asano R Ikeda M. Cardiac lipoma originating from the left ventricular apex diagnosed using the magnetic resonance imaging fat suppression technique: report of a case. Surg Today. (2010) 40:871–3. 10.1007/s00595-009-4146-y 20740352

[17] Kawarai S Yaginuma GY Abe K Hamasaki A Ishikawa K Tanaka D. Left ventricular lipoma with pseudoaneurysm-like appearance. Gen Thorac Cardiovasc Surg. (2010) 58:279–82. 10.1007/s11748-009-0528-8 20549457

[18] Kim SW Hong JM Kim DW. Left ventricular apical lipoma resected under the guidance of a mediastinoscope. Ann Thorac Surg. (2010) 90:1019–21. 10.1016/j.athoracsur.2009.12.030 20732543

[19] Valenti V Zia MI Uretsky S Wolff SD. Intra-myocardial left ventricular lipoma associated with non-compaction cardiomyopathy. Eur Heart J Cardiovasc Imaging. (2012) 13:963. 10.1093/ehjci/jes110 22645205

[20] Patris V Argiriou M Lama N Sakellaridis T Charitos C. Trans-aortic excision of intraventricular lipoma with the assistance of arthroscopic camera. J Thorac Dis. (2013) 5:E140–3. 10.1016/j.ijscr.2012.09.004 23991324 PMC3755683

[21] Steger CM. Intrapericardial giant lipoma displacing the heart. ISRN Cardiol. (2011) 2011:243637. 10.5402/2011/243637 22347636 PMC3262495

[22] Ragland MM Tak T. The role of echocardiography in diagnosing space-occupying lesions of the heart. Clin Med Res. (2006) 4:22–32. 10.3121/cmr.4.1.22 16595790 PMC1447535

[23] Araji OA Gutierrez-Martin MA Miranda N Barquero JM. Video-assisted cardioscopy for removal of primary left ventricular fibroma. Interact Cardiovasc Thorac Surg. (2010) 10:344–5. 10.1510/icvts.2009.223008 19939851

